# Magnetization of active inclusion bodies: comparison with centrifugation in repetitive biotransformations

**DOI:** 10.1186/s12934-018-0987-7

**Published:** 2018-09-03

**Authors:** Romana Koszagova, Tomas Krajcovic, Klaudia Palencarova-Talafova, Vladimir Patoprsty, Alica Vikartovska, Kristyna Pospiskova, Ivo Safarik, Jozef Nahalka

**Affiliations:** 1 Institute of Chemistry, Centre for Glycomics, Slovak Academy of Sciences, Dubravska Cesta 9, SK-84538 Bratislava, Slovak Republic; 2 Institute of Chemistry, Centre of Excellence for White-green Biotechnology, Slovak Academy of Sciences, Trieda Andreja Hlinku 2, SK-94976 Nitra, Slovak Republic; 3 Regional Centre of Advanced Technologies and Materials, Palacky University, Slechtitelu 27, 783 71 Olomouc, Czech Republic; 4 Department of Nanobiotechnology, Biology Centre, ISB, CAS Na Sadkach 7, 370 05 Ceske Budejovice, Czech Republic

**Keywords:** Active inclusion bodies, Magnetic modification, Recycling in biotransformations, Sialic acid aldolase, UDP–glucose pyrophosphorylase

## Abstract

**Background:**

Physiological aggregation of a recombinant enzyme into enzymatically active inclusion bodies could be an excellent strategy to obtain immobilized enzymes for industrial biotransformation processes. However, it is not convenient to recycle “gelatinous masses” of protein inclusion bodies from one reaction cycle to another, as high centrifugation forces are needed in large volumes. The magnetization of inclusion bodies is a smart solution for large-scale applications, enabling an easier separation process using a magnetic field.

**Results:**

Magnetically modified inclusion bodies of UDP–glucose pyrophosphorylase were recycled 50 times, in comparison, inclusion bodies of the same enzyme were inactivated during ten reaction cycles if they were recycled by centrifugation. Inclusion bodies of sialic acid aldolase also showed good performance and operational stability after the magnetization procedure.

**Conclusions:**

It is demonstrated here that inclusion bodies can be easily magnetically modified by magnetic iron oxide particles prepared by microwave-assisted synthesis from ferrous sulphate. The magnetic particles stabilize the repetitive use of the inclusion bodies 
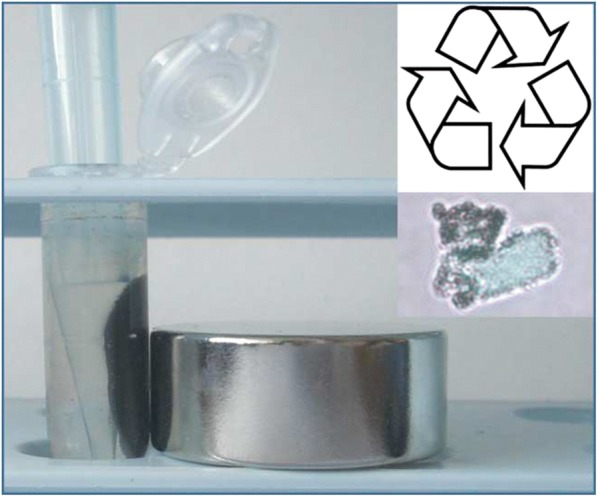
.

**Electronic supplementary material:**

The online version of this article (10.1186/s12934-018-0987-7) contains supplementary material, which is available to authorized users.

## Background

In the biotechnological industry, microbial enzymes are widely used in various biotransformation processes. It is often expensive to use the enzymes from their native hosts; therefore, recombinant enzymes have become a driving force in biotechnology [1]. In view of enzyme recovery, recycling and the improvement of stability, enzyme immobilization techniques were introduced to biotransformation processes [2–4]. Usually, the scientists exploring enzyme expression in prokaryotic hosts are looking for conditions that increase the solubility of the recombinant enzyme [5], and process engineering scientists explore various immobilization techniques that make the enzyme insoluble [2–4]. This paradox can be eliminated when enzyme immobilization is integrated into enzyme overexpression. The integration can be called “in vivo enzyme immobilization” [6, 7]. Inclusion bodies (IBs), which are formed during the overexpression of the enzyme in a prokaryotic host, such as *E. coli*, can be purposely tailored by a fusion of the enzyme with a “pull-down” tag [8]. There are several “pull-down” tags that can be used to produce active enzymes in the form of IBs. The cellulose-binding domain from *Clostridium cellulovorans* was originally used as “pull-down” tag for bioconversion processes [8–10]; however, many proteins induce the formation of active inclusion bodies and can be used as the fusion tag [11, 12]. For example, green fluorescent protein (GFP) has also been used as an *N*-terminal “pull-down” tag [13], and even shorter synthetic peptides have already been designed for “pull-down” or “in vivo enzyme immobilization” [14–16].

The in vivo immobilized enzymes can be directly recovered by centrifugation and applied as carrier-free immobilized enzymes for biocatalysis. The carrier-free immobilized enzymes are particularly valuable when the enzymes have low specific activity [17]. Otherwise, high g-force centrifugation of large volumes is expensive for scale-up processes. To simplify the separation, the entrapment of IBs into alginate beads has been proposed previously [7, 10]. However, the alginate gel generates a diffusion barrier that limits its use in many biotransformations. The preparation and application of magnetically modified IBs, which can be easily separated by a magnetic separator, is described in this paper as a new alternative for the convenient recovery of IBs from the reaction mixture. The magnetic modification procedure at low temperatures [18, 19] is used here and in this way produced “magnetic IBs” that showed excellent operational stability. Three protein examples were magnetically modified: GFP, sialic acid aldolase, and UDP–glucose pyrophosphorylase. It is shown here that in the case of IBs, the slow fixation process in the freezer is not needed or can be substituted by gentle lyophilization. Previously, glucose oxidase had to be crosslinked by glutaraldehyde (Sweetzyme granules) and fixed on magnetic particles in the freezer for a few days (e.g. 7 days) [18, 19].

## Methods

### Materials

Chemically competent *Escherichia coli* BL21(DE3)T1R (CMC0014), CelLytic™ B Cell Lysis Reagent, ferrous sulphate heptahydrate, KOH and other compounds were supplied by Sigma-Aldrich (St. Louis, Missouri, USA). GFP (FPbase: TurboGFP; GenBank: ASW25889), sialic acid aldolase (SAA, UniProt: P0A6L4; NCBI-GeneID: 947742) and UDP–glucose pyrophosphorylase (GalU, UniProt: P0AEP3; NCBI-GeneID: 945730) genes were *N*-terminally fused with the cellulose-binding domain from *Clostridium cellulovorans* by cloning into plasmid pET-34b (Additional file 1).

### Preparation of active inclusion bodies

Chemically competent cells (*Escherichia coli* BL21(DE3)T1R) were transformed with the isolated plasmid and grown on solid LB medium (1.5% agar, 1% peptone, 0.5% yeast extract and 0.5% NaCl) supplemented with kanamycin (30 µg/mL). The selected colonies were transferred to liquid LB medium with kanamycin (30 µg/mL), grown for 24 h at 32 °C, and then inoculated into new medium. After 4 h of cultivation at 37 °C, induction was started with isopropyl β-d-1-thiogalactopyranoside (400 µM), and cells were grown for 24 h at 20 °C. The cells were harvested by centrifugation (4500 *g*, 20 min, 4 °C, 100–250 mL of cultivation broth) and lysed with ten volumes of the non-ionic lytic detergent (CelLytic™). After centrifugation of the lysate (20,000 *g*, 10 min, 4 °C), the debris was washed three times with 10 volumes of 50 mM Tris–HCl buffer, pH 7.8. The insoluble fraction (IBs) was assayed for proteins and enzyme activity as described below. Additional file 1: Figure S4 shows SDS-PAGE of used enzymes in the form of inclusion bodies.

### Preparation of magnetic iron oxides

Upon stirring, a solution of FeSO_4_.7H_2_O (100 mL, 10 g/L) was adjusted to pH 12 by a dropwise addition of 1 M KOH. A brown precipitate of iron hydroxides was formed. After dilution with 100 mL of distilled water in 800-1000 mL beaker, the suspension was treated in a microwave oven at maximum power (960 W) for 10 min. After cooling to room temperature, the formed particles of magnetic iron oxides were repeatedly washed with distilled water until neutral pH was reached; separation during washing was performed using a permanent NdFeB magnet.

### Magnetic modification of active inclusion bodies

The prepared particles of magnetic iron oxides were washed 3 times with 100 mM Tris buffer, pH 8, and separated by a magnet. Tubes were filled with the particle solution and the particles were sedimented by a NdFeB magnet below the bottom of the tube; then, it was diluted with the buffer (supernatant adjusted to four volumes of particles). The particles were supplied with IBs solution (the same Tris buffer, one-quarter volume of the sedimented particles, GFP and SAA experiments − 20 mg protein/mL, GalU and final SAA experiments − 4 mg protein/mL) and the suspension was thoroughly mixed with a pipette (up and down). As is described in the original procedure [18], the suspensions were spread on Petri dishes, the supernatant was carefully removed by a pipette, the Petri dishes were placed into sealed plastic bags with silica desiccant and then left in the freezer for a longer time period to fix the magnetic particles on the surface of enzyme particles (− 20 °C, 3 weeks). In the case of GalU and final SAA experiments, the sediments were frozen directly in tubes (no desiccant) for only 24 h or they were quickly lyophilised (− 10 °C, 1 mBar, 30–60 min, without “the final drying at super-zero temperatures”). Additional file 1: Table S1 shows the specific activities of GalU and final SAA. Comparing the specific activities of free IBs and magnetized IBs, initial mixing with the magnetic particles lowered the activity to approximately 25%.

### Recycling of magnetically modified inclusion bodies in the biotransformation process

After each reaction cycle, the particles were separated by a magnet and supplied with a fresh reaction mixture: SAA experiments, 50 mM ManNAc, 75 mM pyruvate, 50 mM Tris, pH 7.8; GalU experiments, 15 mM glucose-1-phosphate, 10 mM UTP, 100 mM Tris, 30 mM MgCl_2_, pH 8. The reactions were performed at 30 °C with shaking at 250 rpm.

### Capillary electrophoresis

The samples were diluted in buffer (100 mM Tris, 30 mM MgCl_2_, pH 8), cleared/degassed by centrifugation (14,000*g*, 5 min) and analysed by CE (see Additional file 1). The CE was performed on a PrinCE Next/800 system equipped with fused silica capillary 70/30/40. The running buffer was 25 mM sodium tetraborate, pH 9.4. The detector was set at 254 nm (UDP-glucose) or 210 nm (sialic acid).

### Protein content assays

The Total Protein Kit, Micro-Lowry, was used to determine protein concentrations (TP0200-1KT, Sigma-Aldrich). The protein concentration in the IBs was evaluated after their dissolution in 1% SDS.

## Results and discussion

### Magnetic modification of active inclusion bodies

Magnetically responsive biomaterials have been applied in various fields, such as environmental nanotechnology and wastewater treatment [20], tumour tissue visualization and treatment [21], and gene therapy and tissue engineering [22]. Different methods of magnetic particle preparation or magnetic modification [19, 23] are used to provide magnetically responsive materials for different biomedical and environmental applications. Environmental concepts are oriented towards low-cost and easy-to-prepare procedures [24]. The same principle applies to industrial enzyme biotransformations. Moreover, the procedures for magnetic enzyme immobilization should be cheaper than protein isolation and purification procedures employing magnetic materials [25]. As a typical example of magnetic enzyme immobilization, magnetic iron oxide-chitosan [26–28] or magnetic iron oxide-aminosilane [29] supports are prepared first, and then an enzyme is adsorbed or covalently bound if the support is activated by glutaraldehyde [30].

Magnetic iron oxides can be prepared by various synthetic procedures. One of them is based on a simple preparation from the ferrous sulphate using microwave irradiation at high pH [31]. Active IBs represent nanoparticles aggregated by hydrophobic interactions; therefore carriers such as chitosan or aminosilane are not needed. Like whole yeast cells [32], microalgae [33] or even rye straw [34], IBs can be directly modified by magnetic iron oxide particles by mixing appropriate amounts of magnetic iron oxide particles with IBs. It was shown recently that the strong binding of magnetic particles to the target biomaterial that is achieved by a complete drying at elevated temperatures [35] can also be performed by freeze drying [18]. It was observed that commercially immobilized glucose isomerase (Sweetzyme, NovoZyme Corp., granule size 1–3 mm) magnetically modified at − 20 °C was stable in eight repeated reaction cycles with only a negligible decrease in activity over time [18].

**Fig. 1:**
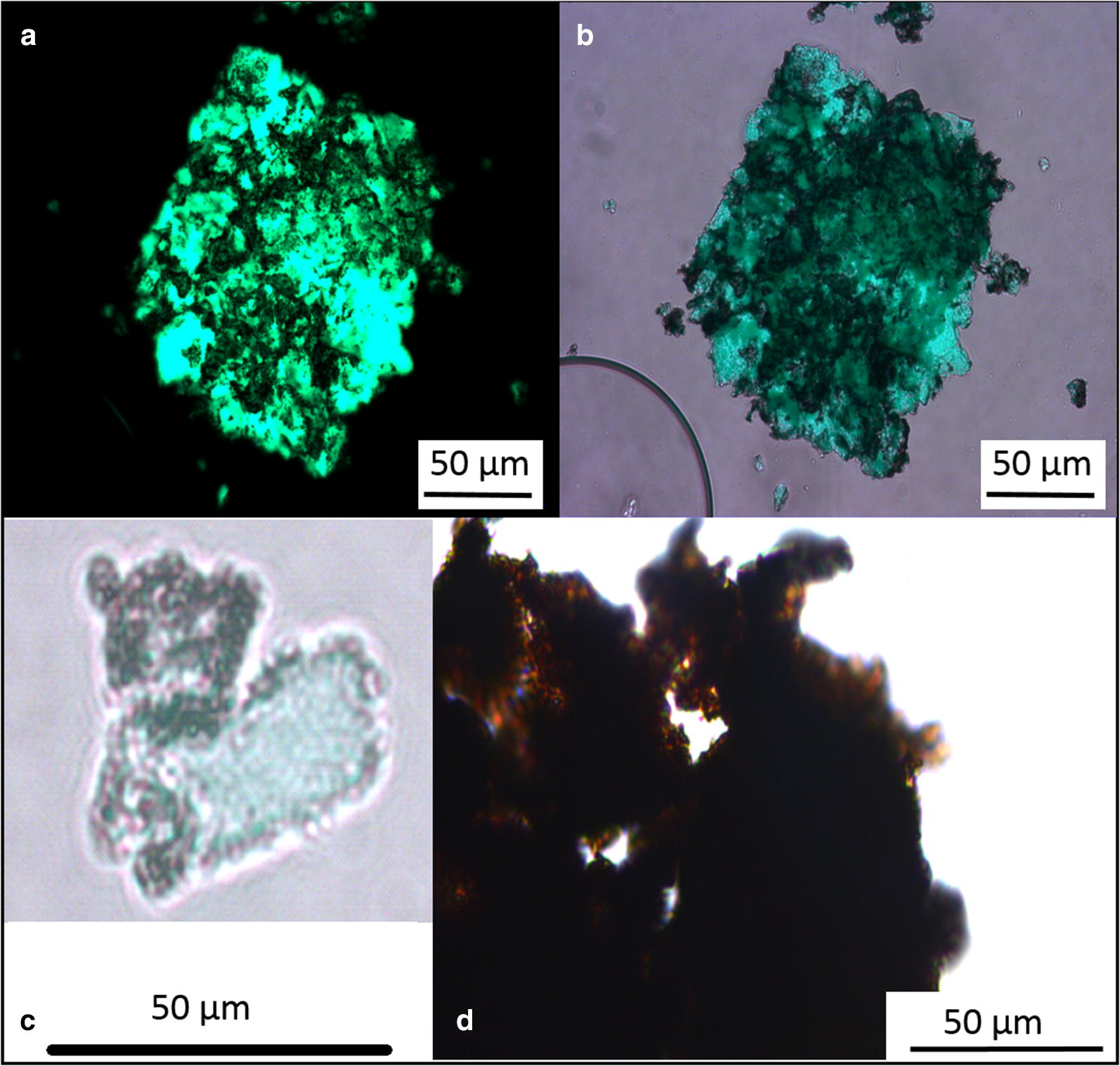
Optical microscopy of magnetized inclusion bodies. **a**–**c** Inclusion bodies of GFP mixed with the magnetic particles in the ratio of 1:1. **a** Fluorescent microscopy image, **b** and **c** light microscopy images. **d** SAA-IBs mixed with the magnetic particles in the ratio of 1:3

In this work, to recycle the active IBs by a magnet, IBs of sialic acid aldolase (SAA-IBs) were chosen as a model enzyme [10]. This enzyme is important for sialic acid synthesis and has potential for various medical applications [36, 37]. However, first, for the microscopic visualization of the magnetic modification procedure, the magnetic iron oxide particles were mixed with IBs of GFP, washed/recycled by buffer/magnet ten times, and then placed in the freezer (see “Methods” section). Microscopic observation after 3 weeks of storage and slow drying in the freezer showed that magnetic particles at a ratio 3 and more completely covered IBs so only black iron oxides were observed; however, when 1 part of the magnetic particle suspension was mixed with 1 part of the GFP-IB suspension, the interaction was well visualized (Fig. 1). Figure 1 depicts how large aggregates of IBs are put together by the magnetic iron oxide particles. They were even still compact after several wash cycles.

The size of IBs that are sphere-like or rod-like in shape [38] is related to the dimensions of the host cell (0.2 to 1.2 μm); however, IBs are clustered into large amorphous aggregates. The aggregation is assumed to be driven by hydrophobic interactions because a generally accepted view is that the formation of IBs is caused by a high local concentration of nascent polypeptides emerging from ribosomes during overexpression, and the cross-interactions among polypeptides do not allow folding of the chain into a regular form, where hydrophobic sequences are oriented inside and hydrophilic sequences outside the overexpressed protein. Surface-exposed hydrophobic sequences are probably the driving force of the interactions. Centrifugation of the cell lysate usually results in the agglutination of IBs to large aggregates. These large aggregates are agglutinated by the magnetic iron oxide particles as shown in Fig. 1. In other words, if the interaction is strong enough, magnetic particles are even well adsorbed on the surface of IBs aggregates (Fig. 1c).

Various forces were considered for protein adsorption onto iron oxide minerals: electrostatic interactions (anion exchange), surface complexation (ligand exchange), hydrophobic interactions, entropic effect, hydrogen bonding, and cation bridging [39]. However, a preferred mode is the surface complexation between protein and iron oxide magnetic particles [39, 40], i.e., a ligand exchange mechanism between surface coordinated hydroxyl groups and water molecules from iron oxides and the proteins. The observation that drying makes the interaction stronger supports this mechanism. On the other hand, in the case of IBs, one can also say that hydrophobic interactions will play an important role.

### Recycling magnetically modified inclusion bodies in the biotransformation process

**Fig. 2:**
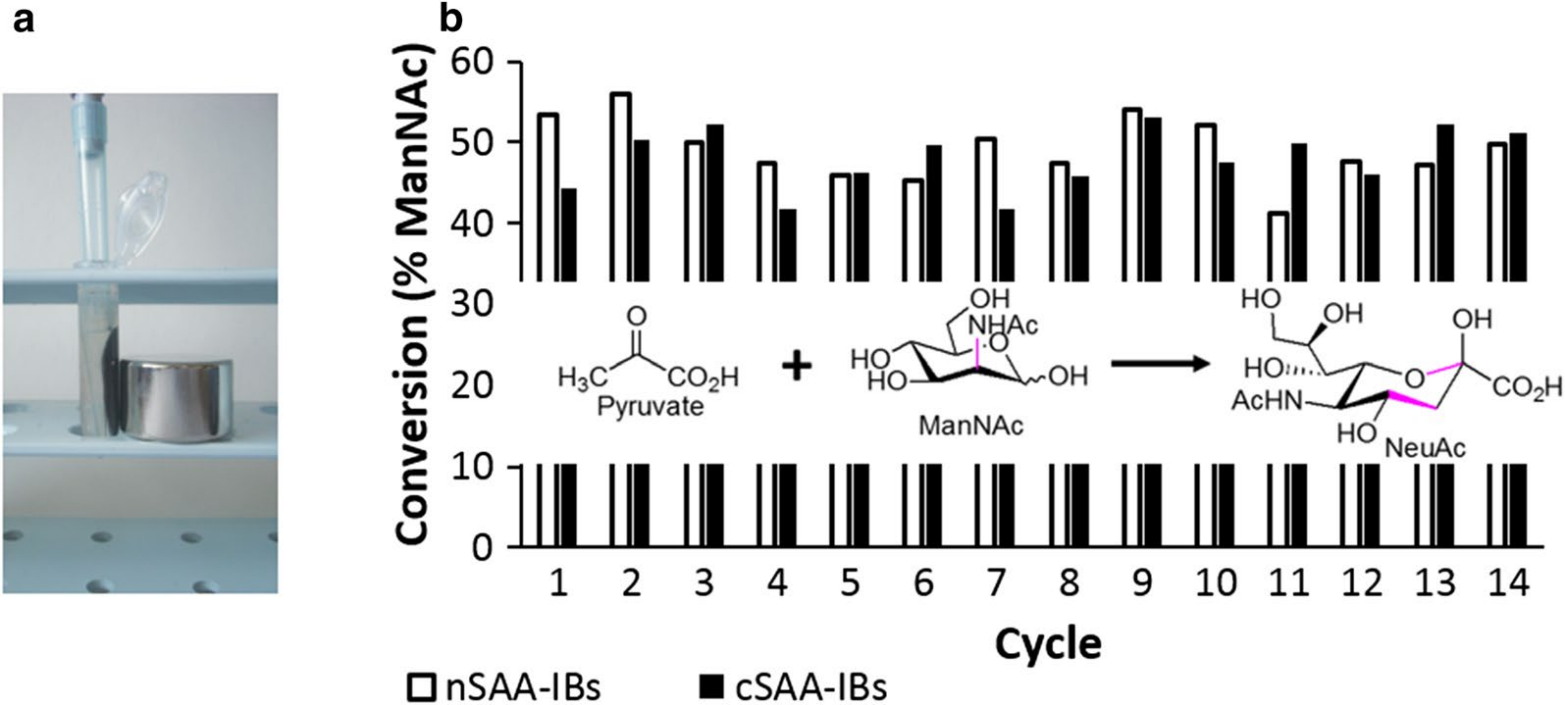
Biotransformations of pyruvate and *N*-acetyl-d-mannosamine (ManNAc) to sialic acid (NeuAc). **a** Inclusion bodies of sialic acid aldolase (SAA-IBs) are easily recycled by a magnet. **b** Non-crosslinked IBs (nSAA-IBs) and crosslinked IBs (cSAA-IBs) in repeated biotransformations, 4 h cycle. Each bar represents the average of two parallel batches

Mixing 3 parts of magnetic particles with 1 part of the SAA-IBs suspension was chosen for the following experiments to ensure that protein leakage into the reaction mixture caused by SAA-IB solubilization will be minimal. The excess of the magnetic particles would physically re-immobilize the enzyme solubilized from IBs during repeated conversions of *N*-acetyl-d-mannosamine (ManNAc) and pyruvate to sialic acid (neuraminic acid, Neu5Ac). It was described previously that Fe_3_O_4_ nanoparticles were able to directly adsorb glucose oxidase (2 mg) when excess magnetic particles (100 mg) have been used [41]. For the possibility of solubilization of IBs and the enzyme release, glutaraldehyde was used for crosslinking in one half of the tubes. It was shown previously [10] that SAA-IBs are still active after a short exposition to aggressive glutaraldehyde crosslinker, so two variants of magnetically modified SAA-IBs were prepared for the comparison: non-crosslinked (nSAA-IBs) and crosslinked (cSAA-IBs). Both variants were easily settled at one side of the plastic tube by a magnet, and the reaction mixture was completely removed and replaced by a fresh one. The process is demonstrated in Fig. 2a. In the reaction, the fixed magnetized SAA-IBs (4.3 mg of protein, particles from two Petri dishes—see materials and methods) were supplied with 1 mL of the reaction mixture (50 mM ManNAc, 75 mM pyruvate, 50 mM Tris). The time course of the first reaction cycle showed that approximately 45% of the ManNAc was converted to sialic acid during the first hour, followed by a slow increase of sialic acid. Four hours were chosen as the reaction interval of the biotransformation cycle (conversion approximately 50%). Figure 2b demonstrates that the biotransformation efficiency was not changed markedly after 14 measured reaction cycles.

IBs represent the protein that is precipitated in “crowded” cytosol conditions; therefore, one cannot expect complete insolubility in many-fold diluted conditions. Slow protein release was expected as mentioned above. Measurement of the protein concentration in the reaction mixtures after transferring the magnetized IBs to a fresh reaction mixture showed that approximately 10 μg/mL was released in each cycle. After fourteen cycles, from the original 4.3 mg of SAA-IBs that was magnetized in both cases, 0.12 mg (2.8%) and 0.11 mg (2.6%) was released from nSAA-IBs and cSAA-IBs, respectively. These values are not negligible. Therefore, in the following experiments, the IBs solutions that were transformed to the magnetic particles were set to five times lower protein concentrations (4 mg/mL). In light of nSAA-IBs and cSAA-IBs comparison, the experiment showed that the crosslinking of IBs is not needed in the presence of magnetic particles. The protein release was similar, probably the magnetic particles re-adsorbed protein released from IBs, and the observed protein leakage was the result of desorption from the magnetic particles.

In this primary study, we decided to focus in the biotransformation process itself. We did not characterize the particles of magnetic iron oxides in terms of individual sizes, and subsequently the particles that are the result of the IBs—modified with the magnetic particles; however, Additional file 1: Figure S3 illustrates the form of magnetite nanoparticles from Fe^2+^ ions in the native state and after fourteen cycles in the biotransformation process (nSAA-IBs).

**Fig. 3:**
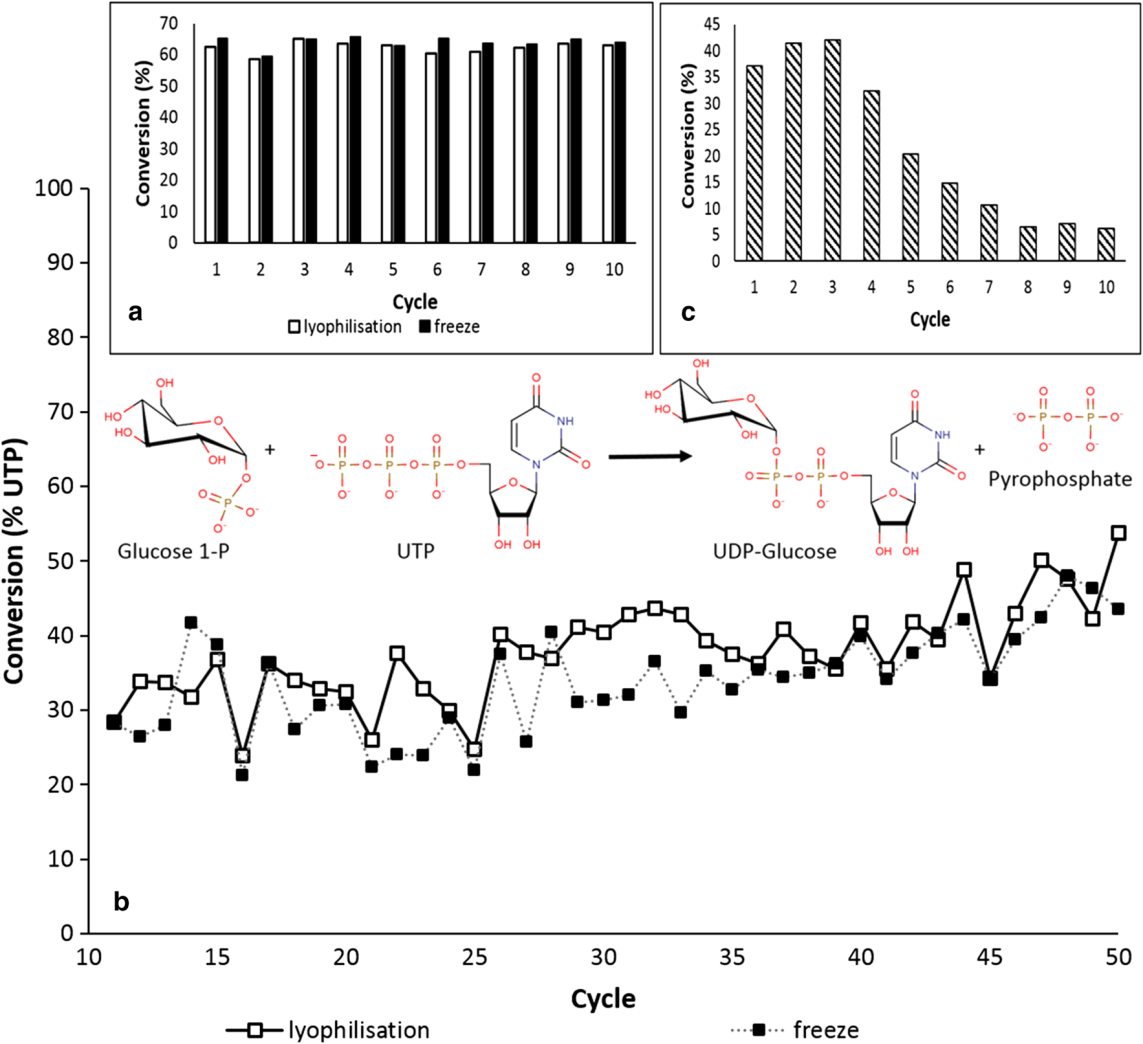
Biotransformations of glucose-1-phosphate and UTP to UDP-glucose and pyrophosphate. **a** Inclusion bodies of UDP–glucose pyrophosphorylase (GalU) recycled by a magnet, 24 h cycle. **b** The same inclusion bodies in 1 h cycle. **c** Inclusion bodies of GalU recycled by centrifugation, 1 h cycle. Each bar represents the average of two parallel batches

Due to the better demonstration of the IBs magnetization procedure, we switched to another enzyme: inclusion bodies of UDP–glucose pyrophosphorylase (GalU). This enzyme catalyses the reversible production of UDP–glucose from Glucose-1-P and UTP, a central compound in glyco-metabolism/glyco-engineering. Active IBs of this enzyme have not been reported previously. In addition, the following experiments were set up to test how the fixation process in the freezer is important because gentle slow drying/fixation in the freezer is not eligible for the scale-up procedure. Magnetized GalU-IBs were frozen directly in the tubes, and one part was lyophilized at conditions without “the final drying at super-zero temperatures”. In the reaction, the magnetized GalU-IBs (100 µL of magnetic particles plus 30 µL of IBs, 0.12 mg of protein) were supplied with 0.5 mL of the reaction mixture (15 mM glucose-1-phosphate, 10 mM UTP, 100 mM Tris, 30 mM MgCl_2_). The results are depicted in Fig. 3. Figure 3a compares lyophilized particles with 24 h frozen particles. There was no difference between them; both type particles reached the maximum degree of conversion in all ten cycles (60%, measured after 24 h of the reaction). After the first ten cycles, the particles were then recycled after 1 hour of the reaction (degree of conversion 30–40%). In comparing the recycling by a magnet with recycling by centrifugation (14,000 rpm, 5 min), tubes with same amount of IBs but without iron oxide particles were set up. Figure 3c shows how GalU-IBs were inactivated during ten reaction cycles. In comparison, magnetically modified particles were recycled fifty times and their activity increased (Fig. 3b, degree of conversion from 30 to 50%). Contrary to free IBs, no proteins were detected in the reaction mixtures after the conversions.

**Fig. 4:**
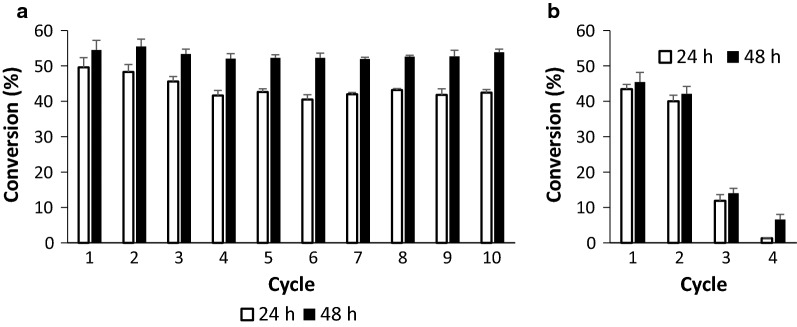
Biotransformations of pyruvate and *N*-acetyl-d-mannosamine (ManNAc) to sialic acid. Inclusion bodies of sialic acid aldolase were lowered five times and the volume of the reaction mixture was increased ten times. **a** Inclusion bodies of sialic acid aldolase (SAA-IBs) recycled by a magnet. **b** Inclusion bodies of SAA recycled by centrifugation. Means and standard errors are shown

In the final experiment, to demonstrate the performance of magnetically modified IBs, 50 mL tubes were filled with 600 µL of magnetic particles mixed with 200 µL SAA-IBs (protein 4 mg/mL), lyophilized and filled with 10 mL of the reaction mixture. In other words, SAA-IBs were lowered five times and the volume of the reaction mixture was increased ten times. At these conditions, the biotransformation was completed after 48 h (Additional file 1: Figure S2). The degree of conversion after 24 h decreased from 51% to 42% during ten cycles, which indicates inactivation; however, the trend after 48 h indicated that many other cycles should be able to perform to the maximum degree of conversion (Fig. 4). It was shown here that biotransformation by IBs adsorbed on magnetic iron oxide particles could run with good performance and operational stability.

## Conclusion

In vivo enzyme immobilization integrates the immobilization procedure into recombinant enzyme production. The physiologically aggregated enzyme can be simply separated from the host cell lysate, washed, and mixed with microparticles consisting of magnetic iron oxides. After gentle lyophilisation, IBs fixed with magnetic iron oxide particles can be repeatedly used in a biotransformation process, and the separation of the magnetically modified biocatalyst from the reaction mixture is easily performed by a magnet. The magnetic modification of active inclusion bodies is a cheap alternative to IBs separation that can be used industrially on a large scale. SAA-IBs that are entrapped in alginate beads and crosslinked by glutaraldehyde have been effectively recycled previously [7, 10]; however, many enzymes are inactivated in the presence of a crosslinker or high concentrations of Ca^2+^ ions. In view of this, the adsorption to magnetic iron oxide particles is a gentler and more general method.

## Additional file

**Additional file 1: Figure S1.** Capillary Electrophoresis, biotransformation of Glc1P and UTP to UDP-Glc and pyrophosphate. **Figure S2.** Capillary Electrophoresis, biotransformation of ManNAc and pyruvate to Sialic acid (NeuAc). 600 µL of magnetic particles mixed with 200 µL SAA-IBs (protein 4 mg/mL), lyophilized and filled with 10 mL of the reaction mixture. **Figure S3.** SEM image of native magnetic particles (A.) and SEM image of magnetic particles plus non-crosslinked IBs of sialic acid aldolase (nSAA-IBs) after 14 repetitive biotransformations (first SAA experiment; B.). **Figure S4.** SDS-PAGE of used enzymes in the form of inclusion bodies. GalU and SAA enzymes are *N*-terminally fused with 20 kDa CBD*clos* - pulldown domain. **Table S1.** Specific activities of used enzymes.

## Abbreviations

IBs: inclusion bodies
GFP: green fluorescent protein
SAA: sialic acid aldolase
GalU: UDP–glucose pyrophosphorylase
GFP-IBs: IBs of GFP
SAA-IBs: IBs of SAA
nSAA-IBs: non-crosslinked SAA-IBs
cSAA-IBs: crosslinked SAA-IBs
ManNAc: *N*-acetyl-d-mannosamine
Neu5Ac: sialic acid
GalU-IBs: IBs of GalU
