# Supplementary material for: Magnetization of active inclusion bodies: comparison with centrifugation in repetitive biotransformations
Source: Microb Cell Fact. 2018 Sep 3;17:139. doi: 10.1186/s12934-018-0987-7 (PMC6122667; doi:10.1186/s12934-018-0987-7)
Supplement: Supplementary file 1 — Additional file 1: Figure S1. Capillary Electrophoresis, biotransformation of Glc1P and UTP to UDP-Glc and pyrophosphate. Figure S2. Capillary Electrophoresis, biotransformation of ManNAc and pyruvate to Sialic acid (NeuAc). 600 µL of magnetic particles mixed with 200 µL SAA-IBs (protein 4 mg/mL), lyophilized and filled with 10 mL of the reaction mixture. Figure S3. SEM image of native magnetic particles (A.) and SEM image of magnetic particles plus non-crosslinked IBs of sialic acid aldolase (nSAA-IBs) after 14 repetitive biotransformations (first SAA experiment; B.). Figure S4. SDS-PAGE of used enzymes in the form of inclusion bodies. GalU and SAA enzymes are N-terminally fused with 20 kDa CBDclos - pulldown domain. Table S1. Specific activities of used enzymes. [file 12934_2018_987_MOESM1_ESM.docx]

Additional file

**Magnetization of active inclusion bodies - comparison with centrifugation in repetitive biotransformations**

Romana Koszagova^1,2^, Tomas Krajcovic^1,2^, Klaudia Palencarova-Talafova^1,2^, Vladimir Patoprsty^1,2^, Alica Vikartovska^1,2^, Kristyna Pospiskova^3^, Ivo Safarik^3,4^, Jozef Nahalka^1,2^*

^1^ Institute of Chemistry, Centre for Glycomics, Slovak Academy of Sciences, Dubravska cesta 9, SK-84538 Bratislava, Slovak Republic

^2^ Institute of Chemistry, Centre of excellence for white-green biotechnology, Slovak Academy of Sciences, Trieda Andreja Hlinku 2, SK-94976 Nitra, Slovak Republic

^3^ Regional Centre of Advanced Technologies and Materials, Palacky University, Slechtitelu 27, 783 71 Olomouc, Czech Republic

^4^ Department of Nanobiotechnology, Biology Centre, ISB, CAS, Na Sadkach 7, 370 05 Ceske Budejovice, Czech Republic

**Cloning**

The genes were amplified from genomic DNA in 50 µL PCR reaction, The target genes with LIC extensions were purified, treated with T4 DNA polymerase for preparation of overhangs, and annealed with the linearized vector pET-34b.

**Capillary electrophoresis**


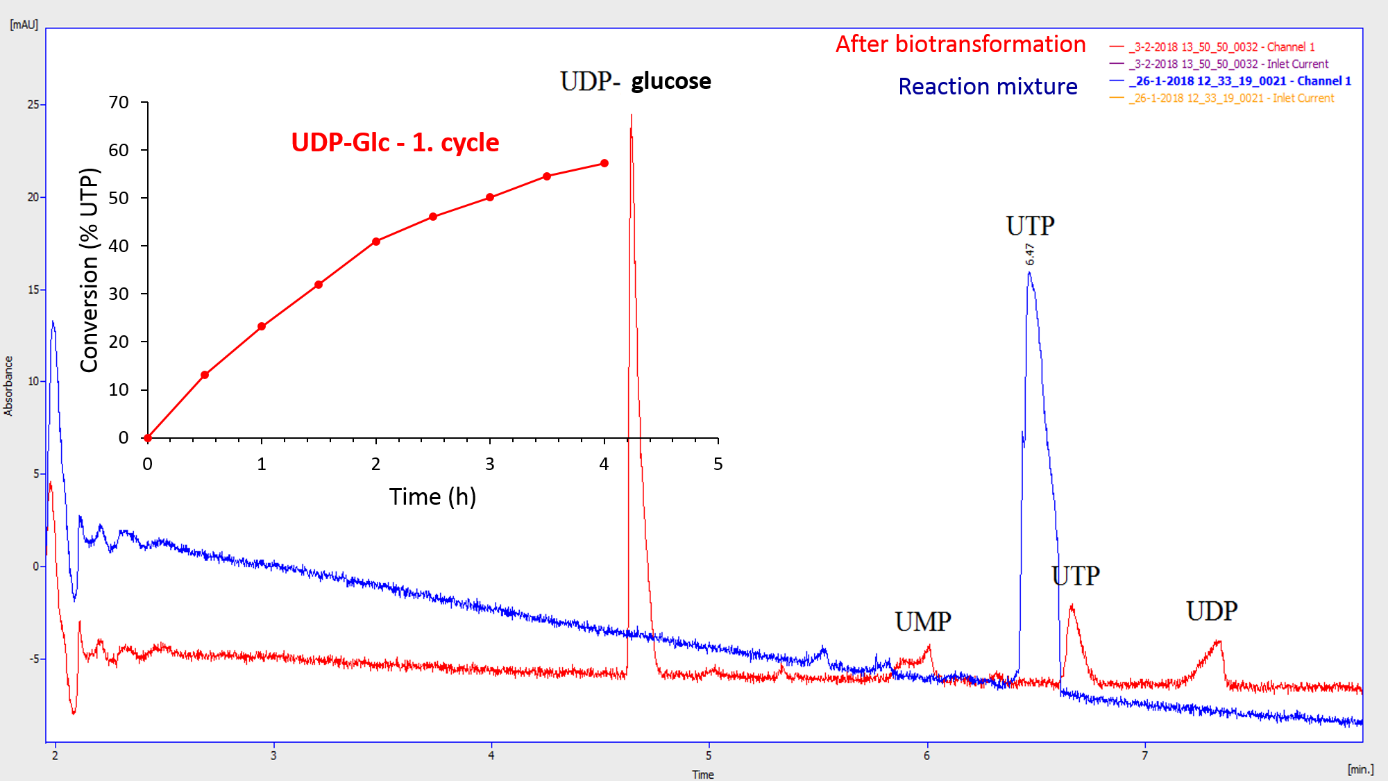


**Figure S1:** Capillary Electrophoresis, biotransformation of Glc1P and UTP to UDP-Glc and pyrophosphate.


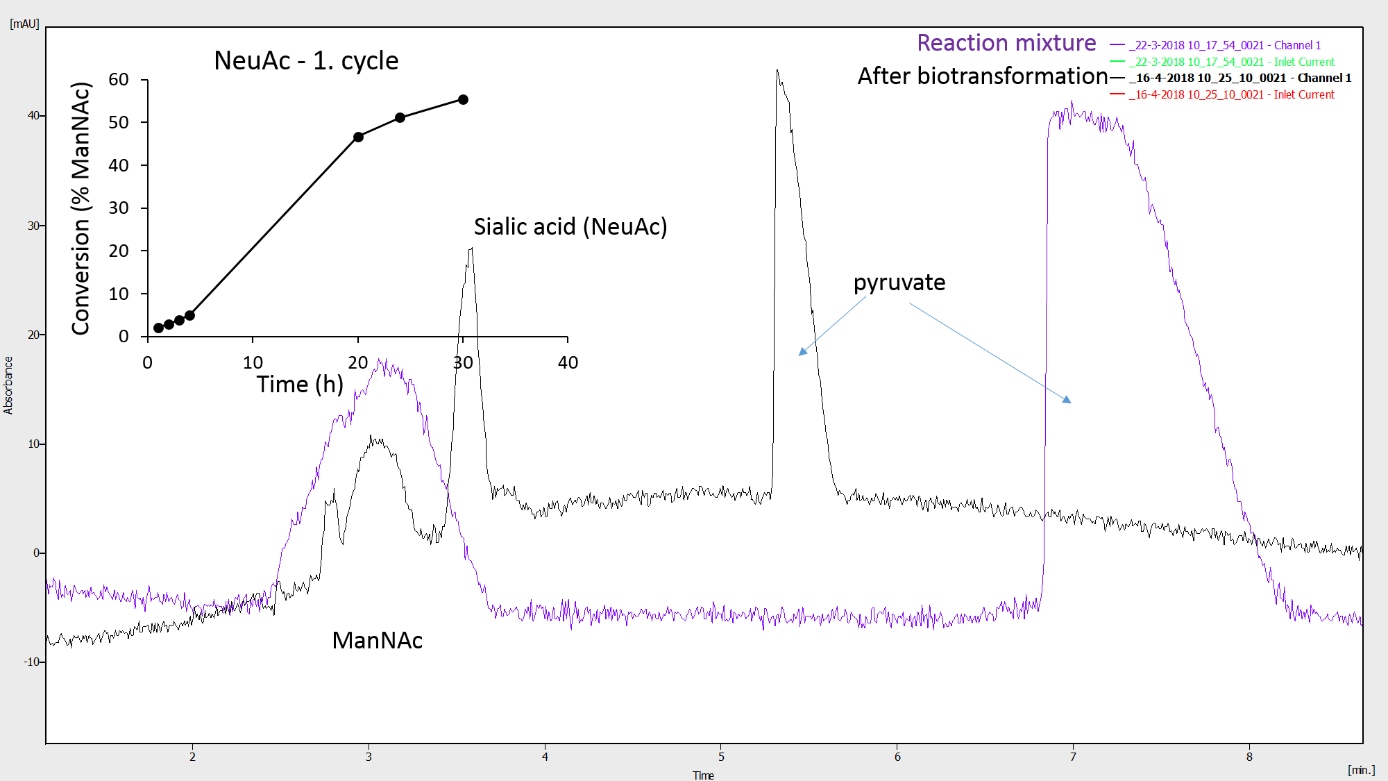


**Figure S2:** Capillary Electrophoresis, biotransformation of ManNAc and pyruvate to Sialic acid (NeuAc). 600 µL of magnetic particles mixed with 200 µL SAA-IBs (protein 4 mg/mL), lyophilized and filled with 10 mL of the reaction mixture.

**SEM image of magnetic particles**


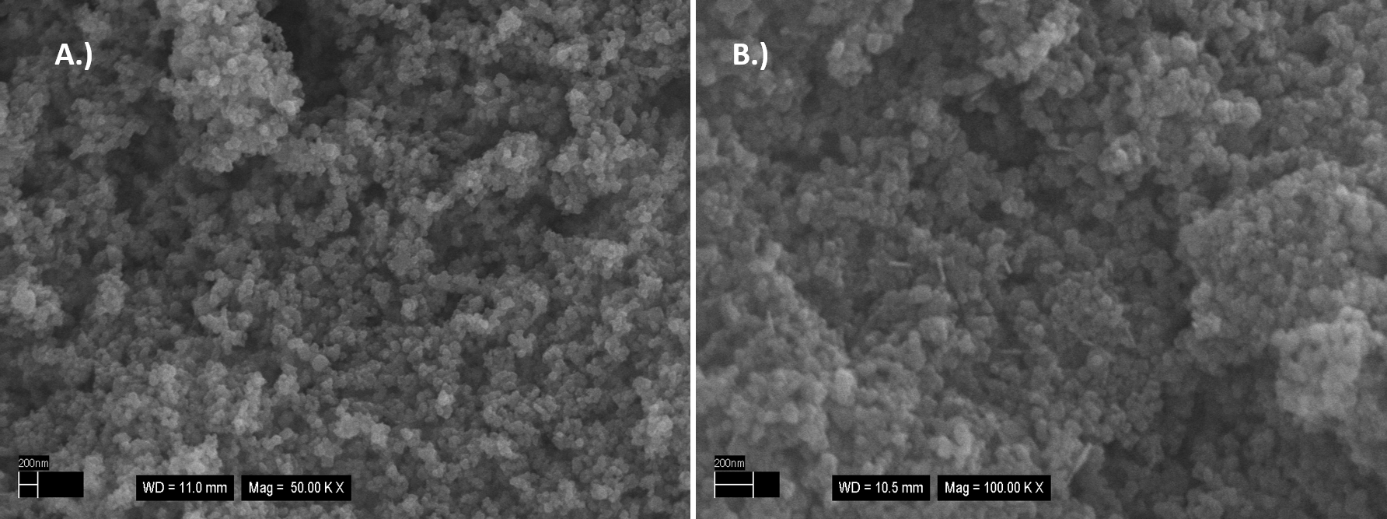


**Figure S3:** SEM image of native magnetic particles (A.) and SEM image of magnetic particles plus non-crosslinked IBs of sialic acid aldolase (nSAA-IBs) after 14 repetitive biotransformations (first SAA experiment; B.).

**Used enzymes**


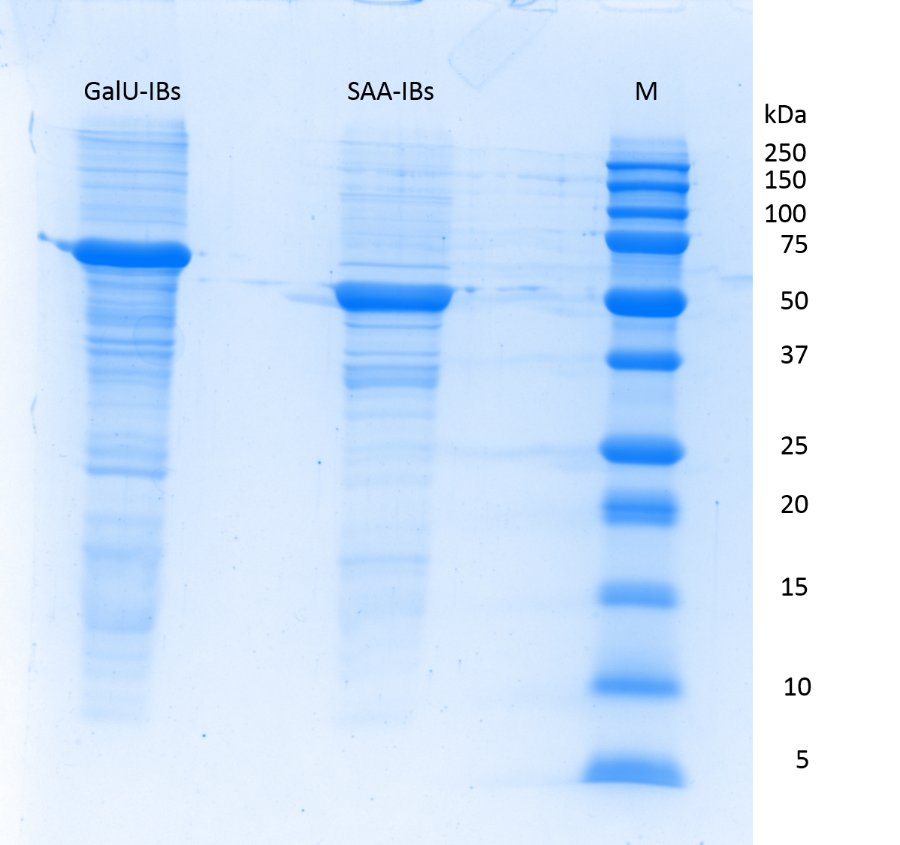


**Figure S4:** SDS-PAGE of used enzymes in the form of inclusion bodies. GalU and SAA enzymes are *N*-terminally fused with 20 kDa CBD*clos* - pulldown domain.

**Table S1:** Specific activities of used enzymes

| IBs | U/mg | % |
| --- | --- | --- |
| SAA-IBs free | 0.667 | 100 |
| SAA-IBs magnetized | 0.178 | 26.68 |
| GalU-IBs free | 0.430 | 100 |
| GalU-Ibs magnetized | 0.107 | 24.83 |
